# Value of Stool-Based Colorectal Cancer Screening: Integrating Real-World Adherence, Detection, and Prevention in a Cohort-Based Modeling Analysis

**DOI:** 10.3390/jcm15010041

**Published:** 2025-12-20

**Authors:** A. Mark Fendrick, Derek W. Ebner, Michael Dore, Chris Estes, Gustavus Aranda, Mohammad Dehghani

**Affiliations:** 1 Division of General Medicine, Department of Internal Medicine, University of Michigan, Ann Arbor, MI 48109, USA; 2 Division of Gastroenterology and Hepatology, Mayo Clinic, Rochester, MN 55902, USA; 3 Department of Medicine, Duke University School of Medicine, Durham, NC 27710, USA; 4 Exact Sciences Corporation, Madison, WI 53719, USA; 5 Department of Mechanical and Industrial Engineering, Northeastern University, Boston, MA 02115, USA

**Keywords:** colorectal cancer, cost, screening, stool test, adherence, sensitivity, specificity

## Abstract

**Background/Objectives:** Modeling analyses for colorectal cancer (CRC) screening focusing solely on the costs of screening do not fully capture the value of screening programs. We evaluated the clinical and economic effects of CRC stool-based screening tests, including impacts on cancer-related outcomes. **Methods:** A cohort-based decision-analytic cost-estimator model estimated outcomes for a single round of screening with next-generation multi-target stool DNA (ng mt-sDNA) test or fecal immunochemical test (FIT) from a US payer perspective. Undiagnosed cancers were assumed to become symptomatic (and detected) within 10 years. Clinical assumptions, advanced precancerous lesion and CRC prevalence, and test performance inputs were from clinical trial data. Adherence rates for initial screening and follow-up colonoscopy after a positive result were from real-world data. Input costs included the screening tests, follow-up colonoscopy (with and without polypectomy), and CRC treatment. **Results:** Compared with FIT, more individuals completed ng mt-sDNA (321,000 vs. 713,000, respectively), leading to the detection of more CRC cases (436 with FIT vs. 2235 with ng mt-sDNA), more advanced precancerous lesions, and more CRC at earlier stages. The cost of screening per patient screened was USD 801 for ng mt-sDNA and USD 124 for FIT. Follow-up colonoscopy cost was USD 149 million with ng mt-sDNA versus USD 22 million with FIT, whereas CRC treatment costs were lower for ng mt-sDNA (USD 1423 million versus USD 1474 million, respectively). When accounting for both direct and CRC averted costs, the total cost of screening and treatment was USD 1383 million with ng mt-sDNA versus USD 1427 million with FIT. **Conclusions:** Higher screening costs with ng mt-sDNA versus FIT are counterbalanced by savings realized from enhanced CRC prevention and earlier detection due to the superior test performance and better adherence with ng mt-sDNA.

## 1. Introduction

Colorectal cancer (CRC) is the third most commonly diagnosed cancer and the second leading cause of cancer-related deaths in the United States, accounting for an estimated 154,000 new cases and 52,900 deaths in 2025 [1]. Despite being largely preventable through screening and removal of precancerous lesions, CRC continues to impose a substantial public health burden. Screening enables both early detection and cancer prevention by identifying advanced precancerous lesions (APLs) and early stage malignancies when curative treatment is more likely [2].

The U.S. Preventive Services Task Force (USPSTF) recommends multiple CRC screening strategies for average-risk adults, including colonoscopy every 10 years, annual fecal immunochemical testing (FIT), and triennial stool DNA–based testing (multi-target stool DNA, mt-sDNA) [3]. The noninvasive stool-based options have expanded access to screening and may improve adherence, particularly among historically underserved populations [4,5]. Despite screening recommendations, uptake remains suboptimal—national adherence rates are approximately 70%, substantially lower than the national target of 80% established by the National Colorectal Cancer Roundtable [6]. Improving adherence and optimizing test choice are therefore key to maximizing the clinical and economic value of CRC screening. Recently, a next-generation mt-sDNA test (ng mt-sDNA) was approved by the U.S. Food and Drug Administration (FDA) for colorectal cancer screening [7]. This test integrates quantitative assessments of DNA methylation and mutation biomarkers, as well as hemoglobin detection, to improve diagnostic accuracy for both APL and early stage CRC compared with the previous mt-sDNA version [8]. While the ng mt-sDNA test and FIT both are stool-based screening tests, they exhibit distinct performance characteristics and cost parameters. Therefore, understanding the comparative economic implications of these different stool-based tests—particularly when real-world adherence, detection rates, and cancer prevention effects are considered—is critical for informing healthcare policy and clinical decision-making. The objective of this analysis was to evaluate the clinical and economic effects of CRC stool-based screening tests, including impacts on cancer-related outcomes.

## 2. Materials and Methods

### 2.1. Model Overview

A cohort-based decision-analytic cost-estimator model was developed to simulate outcomes for 1 million average-risk individuals undergoing a single round of initial screening with either ng mt-sDNA (Cologuard Plus^TM^, Exact Sciences Corporation, Madison, WI, USA) or FIT from a US payer perspective. The model was performed in Microsoft Excel for Microsoft 365 MSO (Version 2511 Build 16.0.19426.20118).

The model included two parallel arms: one in which all individuals were offered ng mt-sDNA, and another in which all were offered FIT. Within each arm, individuals could either complete or not complete the stool-based screening test according to adherence assumptions derived from published data [9,10]. Among those who completed ng mt-sDNA or FIT, outcomes were determined by the test’s performance characteristics and subsequent adherence to diagnostic colonoscopy following a positive result. Individuals with positive ng mt-sDNA or FIT who completed follow-up colonoscopy could have APLs removed (cancer prevention), CRCs detected at an earlier stage (cancer detection), or no lesions found. Individuals who did not undergo follow-up colonoscopy after a positive stool test, or who received a negative stool-based test result despite the presence of an undetected lesion, remained at risk for disease progression.

**Figure 1:**
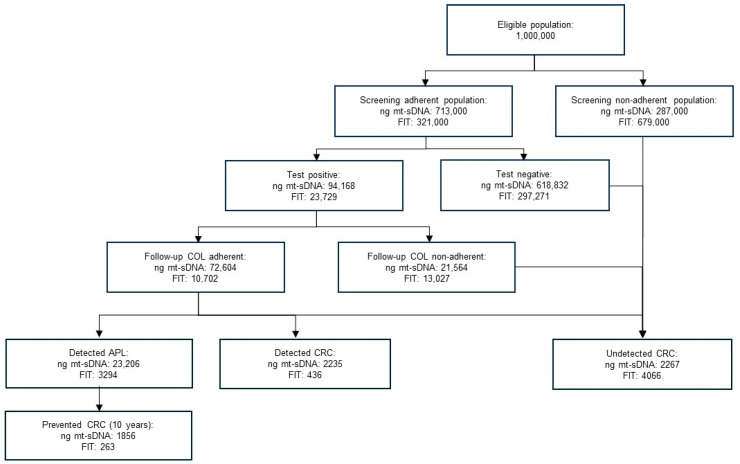
Flowchart of patients through the model. APL—advanced precancerous lesion; COL—colonoscopy; CRC—colorectal cancer; FIT—fecal immunochemical test; ng mt-sDNA—next-generation multi-target stool DNA test.

Undiagnosed cancers were assumed to become symptomatic (and detected) within 10 years, consistent with published natural history data [3,11]. Detection and removal of APLs during follow-up colonoscopy prevented progression to cancer within the same time frame. A schematic overview of the model structure, including adherence pathways and clinical outcome flows, is shown in Figure 1.

### 2.2. Model Inputs

Clinical assumptions, including APL and CRC prevalence, were derived from the summary of safety and effectiveness data (SSED) FDA report (Table 1) [12] of the BLUE-C (NCT04144738) clinical trial for ng mt-sDNA [13]. The FDA creates the SSED reports to standardize population and performance characteristics for approved drugs and medical devices with the same or similar indicated use. The ng mt-sDNA and FIT performance, including the specificity of non-neoplastic findings or negative colonoscopy, and sensitivity of detecting NAA (non-advanced adenoma), APL, and CRC by stage, were also obtained from the SSED FDA report (Table 1) [12]. The performance of a follow-up colonoscopy after a positive stool-based test was the same as that used in the models to inform USPSTF reports (Table 1) [14]. The prevalence of CRC transition from APL in 10 years was from the published literature (Table 1) [15].

Adherence rates for initial screening and follow-up colonoscopy after a positive result were from real-world studies and meta-analysis (71.3% for ng mt-sDNA vs. 32.1% for FIT; and 77.1% vs. 45.1%, respectively, for follow-up colonoscopy after a positive result; Table 1) [9,10,16]. Input costs included the cost of tests [17,18], follow-up colonoscopy (with and without removal of a polyp, including costs for colonoscopy, pathology, anesthesia, and prescription bowel preparation costs) [19,20], and CRC treatment costs [21], with adjustments for survival and inflation [22,23] (Table 1).

**Table 1:** Inputs from clinical trials and the published literature for the economic modeling of colorectal cancer screening.

Category				Value	Reference
Patient population				1,000,000	
Prevalence, %		CRC		0.45	FDA 2024 [12]
		APL		10.4	
		NAA		34.4	
		CRC Transition from APL in 10 years		8	Stryker et al., 1987 [15]
Adherence, %	mt-sDNA	Initial screening		71.3	Le et al., 2025 [9]
		Follow-up colonoscopy		77.1	Greene et al., 2025 [16]
	FIT	Initial screening		32.1	Vahdat et al., 2025 [10]
		Follow-up colonoscopy		45.1	Greene et al., 2025 [16]
Test performance, %	ng mt-sDNA	Sensitivity	CRC Stage I	88.0	FDA 2024 [12]
			CRC Stage II	92.9	
			CRC Stage III	100.0	
			CRC Stage IV	100.0	
			APL	43.3	
			NAA	12.5	
		Specificity	Non-neoplastic findings or negative colonoscopy	92.7	
	FIT	Sensitivity	CRC Stage I	56.0	
			CRC Stage II	78.6	
			CRC Stage III	73.3	
			CRC Stage IV	83.3	
			APL	23.3	
			NAA	6.7	
		Specificity	Non-neoplastic findings or negative colonoscopy	95.7	
	Follow-up colonoscopy	Sensitivity	CRC	95.0	Knudsen et al., 2021 [14]
			APL *	94.0	
Screening costs	ng mt-sDNA	Screening adherent ^†^		USD 591.92	2024 CMS Lab Fee Schedule [18]
	FIT	Screening adherent and non-adherent		USD 18.05	2021 CMS Lab Fee Schedule [17]
	Follow-up colonoscopy	Without polypectomy ^‡^		USD 1602	Fisher et al., 2022 [19]
		With polypectomy ^‡,§^		USD 2223	
CRC treatment costs		Localized		USD 174,362	Fitch et al., 2015 [21]
		Regional		USD 375,526	
		Distant		USD 495,464	

APL: advanced precancerous lesion; CMS: Centers for Medicare and Medicaid Services; CRC: colorectal cancer; FIT: fecal immunochemical test; NAA: non-advanced adenoma; ng mt-sDNA: next-generation mt-sDNA. * Weighted average based on APL prevalence by size and reported performance of colonoscopy: 1.3% lesions < 6 mm (75.0%); 7.4% lesions 6 to <10 mm (85.0%); 91.3% lesions ≥ 10 mm (95.0%) [14,24]. ^†^ ng mt-sDNA price as set by the CMS in November 2024. mt-sDNA only charges for the kits returned. Non-adherent kits are not charged. ^‡^ Based on reported commercial and Medicare costs weighted to US population aged 45–64 and 65–75 years, respectively [19,20]. ^§^ Polypectomy costs applied to neoplastic and non-neoplastic findings requiring biopsy. Based on 4-year CRC treatment costs with adjustments for survival and inflation [22,23].

### 2.3. Outcomes

Clinical outcomes were the numbers of individuals screened, CRC detected, CRC detected at early stages (I or II), APL detected, the number needed to screen to detect a single CRC, and the number of CRC cases prevented over a 10-year horizon through APL detection. Economic outcomes were estimated for a 10-year time horizon from the perspective of a US health care payer and annual discounting was not applied. These outcomes included the total screening costs (including initial screening test cost and cost of follow-up colonoscopy after a positive test), cost of screening per patient screened (total screening costs/number of patients screened), CRC treatment costs (for both CRCs detected by screening or by symptoms, based on the number of CRC detected and by CRC stage), total direct cost (screening plus treatment) per patient screened and by detected CRC, CRC costs saved through prevention by APL detection, and overall total costs (including screening, treatment, and CRC costs saved).

### 2.4. Sensitivity Analysis

The primary sensitivity analysis evaluated overall total costs when FIT screening adherence and follow-up colonoscopy after a positive FIT increased by 20%, 40%, or 60% compared with current real-world adherence values. A second sensitivity analysis evaluated the overall total costs when FIT program costs ranging from USD 20 to USD 100 were incorporated into the model. Finally, a sensitivity analysis was conducted where APL prevalence, CRC prevalence, CRC transition to APL, colonoscopy costs, screening test costs, screening adherence, follow-up colonoscopy adherence, and CRC costs by stage were varied (±10%), and clinical and economic outcomes were estimated.

## 3. Results

With a single round of screening, ng mt-sDNA resulted in more individuals completing the test than FIT (713,000 vs. 321,000, respectively), with more completed follow-up colonoscopies (72,604 vs. 10,702, respectively; Table 2; Figure 1). This higher adherence and differences in test performance (Table 1) for ng mt-sDNA led to more cancers being detected versus FIT (2235 vs. 436, respectively; 413% increase). A relatively higher percentage of early stage (I–II) cancers were detected with ng mt-sDNA than FIT (1016 vs. 191, respectively; 432% more) as compared to later stage (III–IV) cancers (1219 vs. 244, respectively; 400% more). The number needed to screen to detect a single CRC was 319 with ng mt-sDNA versus 737 with FIT, a 57% improvement in screening efficiency (Table 2). Screening with ng mt-sDNA also detected more CRC at earlier stages and more APL than FIT (Table 2). Over a 10-year horizon, the greater number of APLs detected and removed during colonoscopy after a positive ng mt-sDNA translated into 1856 CRC cases averted, compared to 263 cases averted with FIT (Table 2; Figure 1).

The cost of screening per patient screened (including the cost of follow-up colonoscopies) was higher for ng mt-sDNA than FIT (USD 801 vs. USD 124), and the follow-up colonoscopy cost was greater (USD 149 million vs. USD 22 million; Table 2). Importantly, the upfront screening costs were offset by reductions in downstream treatment costs. Even though the number of CRC cases detected were higher in the ng mt-sDNA screened cohort, cancer treatment costs were 3% less with ng mt-sDNA (USD 1423 million) than with FIT (USD 1474 million); the savings were driven by cancer cases being detected at earlier stages. When these treatment savings were incorporated into total costs, the estimated direct cost per screened patient was USD 2797 with ng mt-sDNA and USD 4716 with FIT. Notably, the model projected that an additional USD 612 million in treatment costs were avoided through CRC prevention via APL removal subsequent to screening with ng mt-sDNA compared to US 87 million in costs avoided after FIT screening (Table 2). When accounting for both direct and avoided costs, the overall total cost was USD 1383 million with ng mt-sDNA versus USD 1427 million with FIT, resulting in an overall USD 44 million in savings, despite higher upfront screening investment (Table 2). In the primary sensitivity analysis, FIT screening or follow-up adherence needed to increase by at least 60% to have a lower overall total cost when compared with ng mt-sDNA (Supplemental Figure S1). In the secondary analysis, adding any FIT program costs resulted in a higher overall total cost than ng mt-sDNA (Supplemental Figure S1). When inputs were varied for disease prevalence, adherence, and costs, ng mt-sDNA remained overall cost-saving as compared to FIT. In all scenarios, ng mt-sDNA resulted in more patients screened and greater numbers of detected CRC and APL (Supplemental Table S1).

## 4. Discussion

The true effectiveness of any CRC screening program is demonstrated by the prevention of CRC through the detection and removal of APLs, as well as the detection of cancer at stages when it is more treatable and survival is higher. Key components that impact such effectiveness are real-world adherence to the initial screening test and recommended follow-up colonoscopy and the performance of the indicated tests in detecting APLs and CRC. As such, comparative analyses that focus solely on detection costs (i.e., cost per cancer detected) do not capture the full picture of a screening test’s clinical and economic value and will always favor lower-cost, lower-performance tests. For example, a study that evaluated the screening costs per relevant target finding (i.e., CRC, APL, or sessile serrated polyp ≥1 cm) among stool-based tests found that ng mt-sDNA had a higher cost per CRC case detected than FIT [25]. Our analyses also demonstrated that ng mt-sDNA had higher upfront program expense than FIT. The greater screening costs were offset by savings realized from improved CRC prevention (from polypectomy) and earlier CRC detection due to higher adherence rates [9,10,26] and performance characteristics [12]. Thus, when both screening and treatment are considered, the more expensive screening strategy yielded both superior clinical (cases prevented and detected at earlier stages) and financial outcomes. As such, any analysis that measures only detection costs and does not include population-based health benefits is inherently limited and may misguide practice, policy, and coverage decisions.

The implications of our modeling of a one-time screening event to reflect real-world decision-making situations—such as insurance programs, gap-closure initiatives, or transient patient populations—are limited, as individuals may undergo multiple rounds of screening and may not remain continuously enrolled over many years. The analysis does not reflect cost-effectiveness, quality-adjusted life years, or survival over a full lifetime. However, this short-term perspective provides insight into the immediate value of screening tests when long-term adherence or follow-up cannot be guaranteed. Modeling analyses assessing the long-term impact of stool-based screening for average-risk individuals between the recommended ages of 45–75 years found that triennial ng mt-sDNA had an overall better benefit-to-burden ratio (i.e., life-years gained vs. number of total colonoscopies) than annual FIT [27]. As seen with the current study, the greater long-term benefit-to-burden of ng mt-sDNA over FIT was attributed to its performance (cumulative sensitivity and specificity) [27]. Moreover, previous analyses have demonstrated that over a lifetime and with repeated screening, mt-sDNA every three years is cost-effective for Medicare and commercial payers [28,29].

This analysis is subject to some limitations, namely, inputs were not adjusted for age, sex, race, or socioeconomic status, factors which are known to impact clinical outcomes and adherence. In addition, the analysis was only a partial economic evaluation, without incremental cost-effectiveness ratio or quality-adjusted life year estimation. While data for the natural history progression from precancerous lesions to CRC is lacking, some evidence suggests that progression and growth of polyps may be faster than indicated by the 8% transition to CRC from APL used in this analysis (see refs. [30,31,32]), meaning that the potential clinical and cost benefits to ng mt-sDNA screening are even greater. Furthermore, coding for the model is proprietary, but with the reported parameters and inputs, the analysis can be reproduced or re-analyzed using alternative inputs. Finally, costs of colonoscopy complications and associated patient burden were not included as part of the analysis, and the inclusion of these complications could alter the potential benefits of follow-up colonoscopy after a positive stool test.

Our modeling analyses incorporated both the costs and benefits of a single round of CRC screening for a simulated US cohort. Under the specific assumptions and data sources used for inputs in the model, the results indicate that accelerating recent trends in the use of ng mt-sDNA (increasing) versus FIT (decreasing) would yield favorable clinical and economic outcomes.

## Figures and Tables

**Table 2:** Estimated clinical and economic outcomes from screening 1 million individuals with ng mt-sDNA and FIT with real-world adherence assumptions.

Outcome			FIT	ng mt-sDNA	Delta, ng mt-sDNA vs. FIT (%)
Clinical outcomes	Population	Patient screened	321,000	713,000	+392,000 (122%)
		Patients with follow-up colonoscopy completed	10,702	72,604	+61,902 (578%)
	CRC detection	Patients with detected CRC	436	2235	+1799 (413%)
		Stage I	107	639	+532 (497%)
		Stage II	84	377	+293 (349%)
		Stage III	168	871	+703 (418%)
		Stage IV	76	348	+272 (356%)
		Number to screen to detect one CRC	737	319	−418 (−57%)
	CRC prevention	Patients with detected APL	3294	23,206	+19,913 (605%)
		CRC prevented through APL detection over 10 years	263	1856	+1593 (605%)
Economic outcomes	Screening cost	Cost of screening by stool-test	USD 18 M	USD 422 M	+USD 404 M (2238%)
		Cost of follow-up colonoscopy after positive stool-test	USD 22 M	USD 149 M	+USD 127 M (582%)
		Total screening cost (initial and follow-up colonoscopy)	USD 40 M	USD 571 M	+USD 531 M (1331%)
		Cost of screening per patient screened	USD 124	USD 801	+USD 676 (544%)
	Treatment cost	Total CRC treatment cost *	USD 1474 M	USD 1423 M	−USD 50 M (−3%)
		Total direct CRC cost (screening and treatment) per patient screened	USD 4716	USD 2797	−USD 1919 (−41%)
		Total direct CRC cost (screening and treatment) per detected CRC ^†^	USD 3,474,379	USD 892,358	−USD 2,582,020 (−74%)
	Prevented cost	CRC cost saved (CRC cost prevented through APL detection)	−USD 87 M	−USD 612 M	−USD 525 M (605%)
	Overall cost	Total direct cost (screening and treatment) and avoided (CRC prevented) cost	USD 1427 M	USD 1383 M	−USD 44 M (−3%)

APL: advanced precancerous lesion; CRC: colorectal cancer; FIT: fecal immunochemical test; ng mt-sDNA: next-generation mt-sDNA. * Based on number of CRC detected and CRC stage. ^†^ Total direct cost divided by detected CRC for each modality (ng mt-sDNA: USD 1994 M/2235 cases; FIT: USD 1514 M/436 cases).

## Data Availability

The original contributions presented in this study are included in the article. Further inquiries can be directed to the corresponding author.

## Supplementary Materials

The following supporting information can be downloaded at https://www.mdpi.com/article/10.3390/jcm15010041/s1, Figure S1: Total cost difference—FIT vs. ng mt-sDNA, with varied FIT adherence and FIT cost inputs. Table S1. Sensitivity Analysis: Estimated clinical and economic outcomes from screening 1 million individuals with ng mt-sDNA and FIT with real-world adherence assumptions.

## Abbreviations

The following abbreviations are used in this manuscript:

APL	Advanced precancerous lesions
CRC	Colorectal cancer
FDA	US Food and Drug Administration
FIT	Fecal immunochemical test
NAA	Non-advanced adenoma
ng mt-sDNA	Next-generation multi-target stool DNA
SSED	Summary of safety and effectiveness data
USPSTF	U.S. Preventative Services Task Force
