# Supplementary material for: Value of Stool-Based Colorectal Cancer Screening: Integrating Real-World Adherence, Detection, and Prevention in a Cohort-Based Modeling Analysis
Source: J Clin Med. 2025 Dec 20;15(1):41. doi: 10.3390/jcm15010041 (PMC12786559; doi:10.3390/jcm15010041)
Supplement: Supplementary file 1 [file jcm-15-00041-s001.zip › jcm-4026595-supplementary.pdf]

Supplemental Material

Figure S1. Total cost difference – FIT vs ng mt-sDNA with varied FIT adherence and FIT cost inputs.

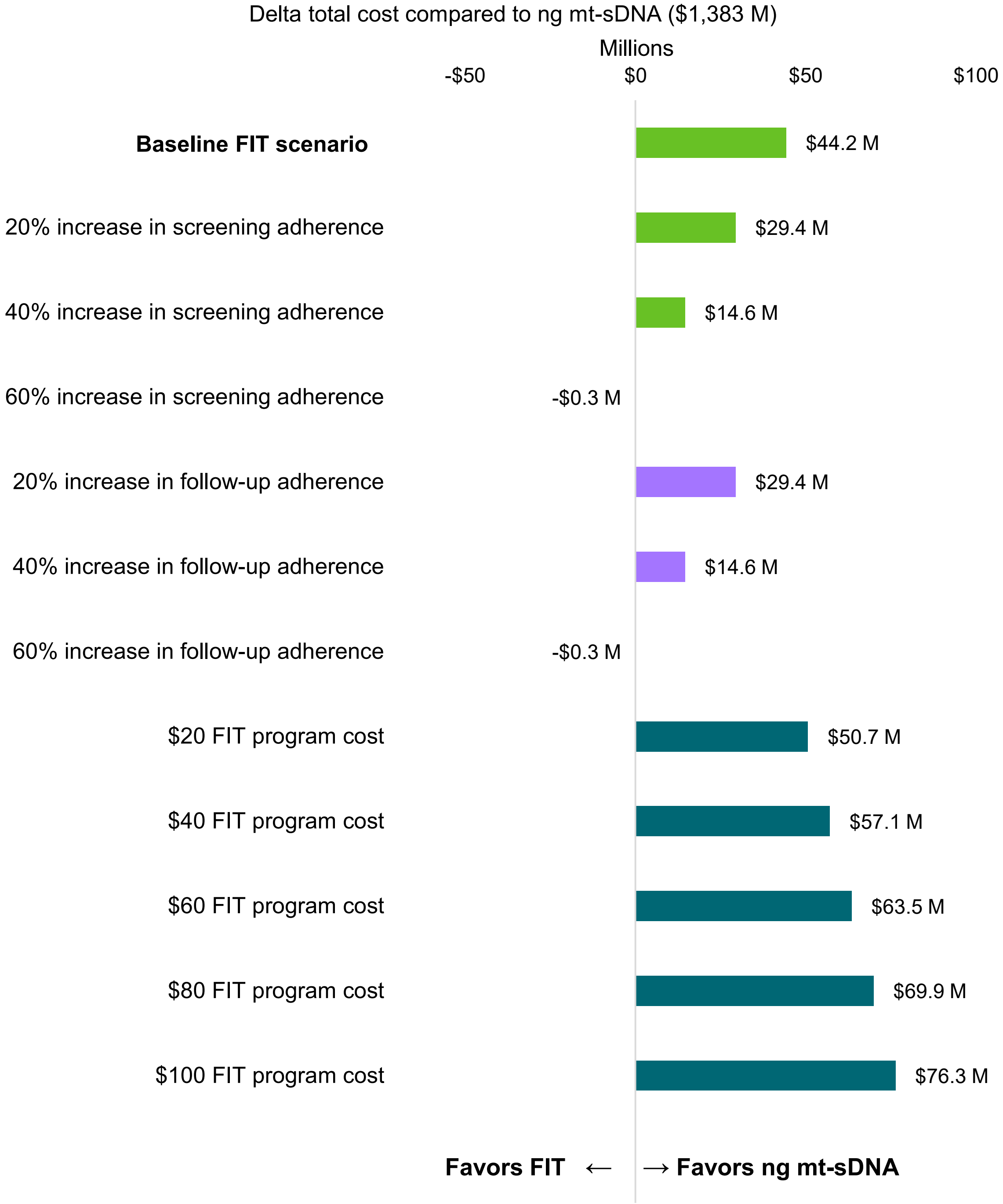

Table S1. Sensitivity Analysis: Estimated clinical and economic outcomes from screening 1 million individuals with ng mt-sDNA and FIT with real-world adherence assumptions.

Category	Base-case value	Threshold value	Population - Patients screened	Population - Patients with follow-up colonoscopy completed	CRC detection - Patients with detected CRC	CRC prevention - Patients with detected APL	Overall cost - Total direct cost and opportunistic (CRC prevented) cost
APL prevalence	10.4%	11.4% (+10%)	324,332 (FIT)<br>720,401 (ng mt-sDNA)<br>+396,069 (122%)	11,052 (FIT)<br>75,072 (ng mt-sDNA)<br>+64,020 (579%)	436 (FIT)<br>2,235 (ng mt-sDNA)<br>+1,799 (413%)	3,623 (FIT)<br>25,527 (ng mt-sDNA)<br>+21,904 (605%)	$1,419 M (FIT)<br>$1,331 M (ng mt-sDNA)<br>-$88 M (-6%)
		9.3% (-10%)	317,668 (FIT)<br>705,599 (ng mt-sDNA)<br>+387,931 (122%)	10,352 (FIT)<br>70,135 (ng mt-sDNA)<br>+59,783 (578%)	436 (FIT)<br>2,235 (ng mt-sDNA)<br>+1,799 (413%)	2,964 (FIT)<br>20,885 (ng mt-sDNA)<br>+17,921 (605%)	$1,435 M (FIT)<br>$1,434 M (ng mt-sDNA)<br>-$1 M (0%)
CRC prevalence	0.45%	0.50% (+10%)	321,145 (FIT)<br>713,321 (ng mt-sDNA)<br>+392,176 (122%)	10,748 (FIT)<br>72,839 (ng mt-sDNA)<br>+62,091 (578%)	479 (FIT)<br>2,458 (ng mt-sDNA)<br>+1,979 (413%)	3,294 (FIT)<br>23,206 (ng mt-sDNA)<br>+19,913 (605%)	$1,574 M (FIT)<br>$1,526 M (ng mt-sDNA)<br>-$49 M (-3%)
		0.41% (-10%)	320,855 (FIT)<br>712,679 (ng mt-sDNA)<br>+391,824 (122%)	10,656 (FIT)<br>72,368 (ng mt-sDNA)<br>+61,712 (579%)	392 (FIT)<br>2,011 (ng mt-sDNA)<br>+1,619 (413%)	3,294 (FIT)<br>23,206 (ng mt-sDNA)<br>+19,913 (605%)	$1,279 M (FIT)<br>$1,240 M (ng mt-sDNA)<br>-$40 M (-3%)
Colonoscopy cost with polypectomy	$ 2,223	$2,445 (+10%)	321,000 (FIT)<br>713,000 (ng mt-sDNA)<br>+392,000 (122%)	10,702 (FIT)<br>72,604 (ng mt-sDNA)<br>+61,902 (578%)	436 (FIT)<br>2,235 (ng mt-sDNA)<br>+1,799 (413%)	3,294 (FIT)<br>23,206 (ng mt-sDNA)<br>+19,913 (605%)	$1,429 M (FIT)<br>$1,394 M (ng mt-sDNA)<br>-$34 M (-2%)
		$2,000 (-10%)	321,000 (FIT)<br>713,000 (ng mt-sDNA)<br>+392,000 (122%)	10,702 (FIT)<br>72,604 (ng mt-sDNA)<br>+61,902 (578%)	436 (FIT)<br>2,235 (ng mt-sDNA)<br>+1,799 (413%)	3,294 (FIT)<br>23,206 (ng mt-sDNA)<br>+19,913 (605%)	$1,425 M (FIT)<br>$1,371 M (ng mt-sDNA)<br>-$54 M (-4%)

Colonoscopy cost without polypectomy	$ 1,602	$1,762 (+10%)	321,000 (FIT)<br>713,000 (ng mt-sDNA)<br>+392,000 (122%)	10,702 (FIT)<br>72,604 (ng mt-sDNA)<br>+61,902 (578%)	436 (FIT)<br>2,235 (ng mt-sDNA)<br>+1,799 (413%)	3,294 (FIT)<br>23,206 (ng mt-sDNA)<br>+19,913 (605%)	$1,427 M (FIT)<br>$1,386 M (ng mt-sDNA)<br>-$42 M (-3%)
		$1,442 (-10%)	321,000 (FIT)<br>713,000 (ng mt-sDNA)<br>+392,000 (122%)	10,702 (FIT)<br>72,604 (ng mt-sDNA)<br>+61,902 (578%)	436 (FIT)<br>2,235 (ng mt-sDNA)<br>+1,799 (413%)	3,294 (FIT)<br>23,206 (ng mt-sDNA)<br>+19,913 (605%)	$1,426 M (FIT)<br>$1,379 M (ng mt-sDNA)<br>-$47 M (-3%)
FIT cost	$ 18.05	$19.86 (+10%)	321,000 (FIT)<br>713,000 (ng mt-sDNA)<br>+392,000 (122%)	10,702 (FIT)<br>72,604 (ng mt-sDNA)<br>+61,902 (578%)	436 (FIT)<br>2,235 (ng mt-sDNA)<br>+1,799 (413%)	3,294 (FIT)<br>23,206 (ng mt-sDNA)<br>+19,913 (605%)	$1,429 M (FIT)<br>$1,383 M (ng mt-sDNA)<br>-$46 M (-3%)
		$16.25 (-10%)	321,000 (FIT)<br>713,000 (ng mt-sDNA)<br>+392,000 (122%)	10,702 (FIT)<br>72,604 (ng mt-sDNA)<br>+61,902 (578%)	436 (FIT)<br>2,235 (ng mt-sDNA)<br>+1,799 (413%)	3,294 (FIT)<br>23,206 (ng mt-sDNA)<br>+19,913 (605%)	$1,425 M (FIT)<br>$1,383 M (ng mt-sDNA)<br>-$42 M (-3%)
CG cost	$ 591.92	$651.11 (+10%)	321,000 (FIT)<br>713,000 (ng mt-sDNA)<br>+392,000 (122%)	10,702 (FIT)<br>72,604 (ng mt-sDNA)<br>+61,902 (578%)	436 (FIT)<br>2,235 (ng mt-sDNA)<br>+1,799 (413%)	3,294 (FIT)<br>23,206 (ng mt-sDNA)<br>+19,913 (605%)	$1,427 M (FIT)<br>$1,425 M (ng mt-sDNA)<br>-$2 M (0%)
		$532.73 (-10%)	321,000 (FIT)<br>713,000 (ng mt-sDNA)<br>+392,000 (122%)	10,702 (FIT)<br>72,604 (ng mt-sDNA)<br>+61,902 (578%)	436 (FIT)<br>2,235 (ng mt-sDNA)<br>+1,799 (413%)	3,294 (FIT)<br>23,206 (ng mt-sDNA)<br>+19,913 (605%)	$1,427 M (FIT)<br>$1,340 M (ng mt-sDNA)<br>-$86 M (-6%)
Adherence to CG	71%	78.4% (+10%)	321,000 (FIT)<br>784,300 (ng mt-sDNA)<br>+463,300 (144%)	10,702 (FIT)<br>79,864 (ng mt-sDNA)<br>+69,162 (646%)	436 (FIT)<br>2,458 (ng mt-sDNA)<br>+2,023 (464%)	3,294 (FIT)<br>25,527 (ng mt-sDNA)<br>+22,233 (675%)	$1,427 M (FIT)<br>$1,373 M (ng mt-sDNA)<br>-$54 M (-4%)
		64.2% (-10%)	321,000 (FIT)<br>641,700 (ng mt-sDNA)<br>+320,700 (100%)	10,702 (FIT)<br>65,343 (ng mt-sDNA)<br>+54,641 (511%)	436 (FIT)<br>2,011 (ng mt-sDNA)<br>+1,576 (362%)	3,294 (FIT)<br>20,885 (ng mt-sDNA)<br>+17,592 (534%)	$1,427 M (FIT)<br>$1,393 M (ng mt-sDNA)<br>-$34 M (-2%)

Adherence to FIT	32%	35.3% (+10%)	353,100 (FIT)<br>713,000 (ng mt-sDNA)<br>+359,900 (102%)	11,772 (FIT)<br>72,604 (ng mt-sDNA)<br>+60,831 (517%)	479 (FIT)<br>2,235 (ng mt-sDNA)<br>+1,756 (366%)	3,623 (FIT)<br>23,206 (ng mt-sDNA)<br>+19,583 (541%)	$1,419 M (FIT)<br>$1,383 M (ng mt-sDNA)<br>-$37 M (-3%)
		28.9% (-10%)	288,900 (FIT)<br>713,000 (ng mt-sDNA)<br>+424,100 (147%)	9,632 (FIT)<br>72,604 (ng mt-sDNA)<br>+62,972 (654%)	392 (FIT)<br>2,235 (ng mt-sDNA)<br>+1,843 (470%)	2,964 (FIT)<br>23,206 (ng mt-sDNA)<br>+20,242 (683%)	$1,434 M (FIT)<br>$1,383 M (ng mt-sDNA)<br>-$52 M (-4%)
Adherence to FU COL CG	77%	84.8% (+10%)	321,000 (FIT)<br>713,000 (ng mt-sDNA)<br>+392,000 (122%)	10,702 (FIT)<br>79,864 (ng mt-sDNA)<br>+69,162 (646%)	436 (FIT)<br>2,458 (ng mt-sDNA)<br>+2,023 (464%)	3,294 (FIT)<br>25,527 (ng mt-sDNA)<br>+22,233 (675%)	$1,427 M (FIT)<br>$1,330 M (ng mt-sDNA)<br>-$96 M (-7%)
		69.4% (-10%)	321,000 (FIT)<br>713,000 (ng mt-sDNA)<br>+392,000 (122%)	10,702 (FIT)<br>65,343 (ng mt-sDNA)<br>+54,641 (511%)	436 (FIT)<br>2,011 (ng mt-sDNA)<br>+1,576 (362%)	3,294 (FIT)<br>20,885 (ng mt-sDNA)<br>+17,592 (534%)	$1,427 M (FIT)<br>$1,435 M (ng mt-sDNA)<br>+$8 M (1%)
Adherence to FU COL FIT	45%	49.6% (+10%)	321,000 (FIT)<br>713,000 (ng mt-sDNA)<br>+392,000 (122%)	11,772 (FIT)<br>72,604 (ng mt-sDNA)<br>+60,831 (517%)	479 (FIT)<br>2,235 (ng mt-sDNA)<br>+1,756 (366%)	3,623 (FIT)<br>23,206 (ng mt-sDNA)<br>+19,583 (541%)	$1,419 M (FIT)<br>$1,383 M (ng mt-sDNA)<br>-$37 M (-3%)
		40.6% (-10%)	321,000 (FIT)<br>713,000 (ng mt-sDNA)<br>+392,000 (122%)	9,632 (FIT)<br>72,604 (ng mt-sDNA)<br>+62,972 (654%)	392 (FIT)<br>2,235 (ng mt-sDNA)<br>+1,843 (470%)	2,964 (FIT)<br>23,206 (ng mt-sDNA)<br>+20,242 (683%)	$1,434 M (FIT)<br>$1,383 M (ng mt-sDNA)<br>-$52 M (-4%)
CRC cost localized	$ 174,362	$191,798 (+10%)	321,000 (FIT)<br>713,000 (ng mt-sDNA)<br>+392,000 (122%)	10,702 (FIT)<br>72,604 (ng mt-sDNA)<br>+61,902 (578%)	436 (FIT)<br>2,235 (ng mt-sDNA)<br>+1,799 (413%)	3,294 (FIT)<br>23,206 (ng mt-sDNA)<br>+19,913 (605%)	$1,455 M (FIT)<br>$1,403 M (ng mt-sDNA)<br>-$52 M (-4%)
		$156,926 (-10%)	321,000 (FIT)<br>713,000 (ng mt-sDNA)<br>+392,000 (122%)	10,702 (FIT)<br>72,604 (ng mt-sDNA)<br>+61,902 (578%)	436 (FIT)<br>2,235 (ng mt-sDNA)<br>+1,799 (413%)	3,294 (FIT)<br>23,206 (ng mt-sDNA)<br>+19,913 (605%)	$1,399 M (FIT)<br>$1,362 M (ng mt-sDNA)<br>-$37 M (-3%)

CRC cost regional	$ 375,526	$413,079 (+10%)	321,000 (FIT)<br>713,000 (ng mt-sDNA)<br>+392,000 (122%)	10,702 (FIT)<br>72,604 (ng mt-sDNA)<br>+61,902 (578%)	436 (FIT)<br>2,235 (ng mt-sDNA)<br>+1,799 (413%)	3,294 (FIT)<br>23,206 (ng mt-sDNA)<br>+19,913 (605%)	$1,491 M (FIT)<br>$1,422 M (ng mt-sDNA)<br>-$69 M (-5%)
		$337,974 (-10%)	321,000 (FIT)<br>713,000 (ng mt-sDNA)<br>+392,000 (122%)	10,702 (FIT)<br>72,604 (ng mt-sDNA)<br>+61,902 (578%)	436 (FIT)<br>2,235 (ng mt-sDNA)<br>+1,799 (413%)	3,294 (FIT)<br>23,206 (ng mt-sDNA)<br>+19,913 (605%)	$1,363 M (FIT)<br>$1,344 M (ng mt-sDNA)<br>-$19 M (-1%)
CRC cost distant	$ 495,464	$545,010 (+10%)	321,000 (FIT)<br>713,000 (ng mt-sDNA)<br>+392,000 (122%)	10,702 (FIT)<br>72,604 (ng mt-sDNA)<br>+61,902 (578%)	436 (FIT)<br>2,235 (ng mt-sDNA)<br>+1,799 (413%)	3,294 (FIT)<br>23,206 (ng mt-sDNA)<br>+19,913 (605%)	$1,474 M (FIT)<br>$1,405 M (ng mt-sDNA)<br>-$70 M (-5%)
		$445,917 (-10%)	321,000 (FIT)<br>713,000 (ng mt-sDNA)<br>+392,000 (122%)	10,702 (FIT)<br>72,604 (ng mt-sDNA)<br>+61,902 (578%)	436 (FIT)<br>2,235 (ng mt-sDNA)<br>+1,799 (413%)	3,294 (FIT)<br>23,206 (ng mt-sDNA)<br>+19,913 (605%)	$1,380 M (FIT)<br>$1,361 M (ng mt-sDNA)<br>-$19 M (-1%)
APL progression to CRC	8.0%	8.8% (+10%)	321,000 (FIT)<br>713,000 (ng mt-sDNA)<br>+392,000 (122%)	10,702 (FIT)<br>72,604 (ng mt-sDNA)<br>+61,902 (578%)	436 (FIT)<br>2,235 (ng mt-sDNA)<br>+1,799 (413%)	3,294 (FIT)<br>23,206 (ng mt-sDNA)<br>+19,913 (605%)	$1,418 M (FIT)<br>$1,322 M (ng mt-sDNA)<br>-$97 M (-7%)
		7.2% (-10%)	321,000 (FIT)<br>713,000 (ng mt-sDNA)<br>+392,000 (122%)	10,702 (FIT)<br>72,604 (ng mt-sDNA)<br>+61,902 (578%)	436 (FIT)<br>2,235 (ng mt-sDNA)<br>+1,799 (413%)	3,294 (FIT)<br>23,206 (ng mt-sDNA)<br>+19,913 (605%)	$1,436 M (FIT)<br>$1,444 M (ng mt-sDNA)<br>+$8 M (1%)
